# Raising the bar: Recovery ambition for species at risk in Canada and the US

**DOI:** 10.1371/journal.pone.0224021

**Published:** 2019-11-19

**Authors:** Kylee A. Pawluk, Caroline H. Fox, Christina N. Service, Eva H. Stredulinsky, Heather M. Bryan

**Affiliations:** 1 Department of Geography, University of Victoria, Victoria, British Columbia, Canada; 2 Department of Oceanography, Dalhousie University, Halifax, Nova Scotia, Canada; 3 Raincoast Conservation Foundation, Sidney, British Columbia, Canada; 4 Hakai Institute, Heriot Bay, British Columbia, Canada; U.S. Geological Survey, UNITED STATES

## Abstract

Routinely crossing international borders and/or persisting in populations across multiple countries, species are commonly subject to a patchwork of endangered species legislation. Canada and the United States share numerous endangered species; their respective acts, the Species at Risk Act (SARA) and the Endangered Species Act (ESA), require documents that outline requirements for species recovery. Although there are many priorities for improving endangered species legislation effectiveness, species recovery goals are a crucial component. We compared recovery goal quality, as measured by goal quantitativeness and ambition, for species listed under SARA and ESA. By comparing across ESA and SARA, the intent of the study was to identify differences and similarities that could support the development of stronger species’ recovery goals under both legislations. Our results indicated that: (1) overall, only 38% of recovery goals were quantitative, 41% had high ambition, and 26% were both quantitative and with high ambition; (2) recovery goals had higher quantitativeness and ambition under ESA than SARA; (3) recovery goals for endangered species had higher ambition than threatened species under ESA and SARA, and; (4) no recovery goal aimed to restore populations to historic levels. Combined, these findings provide guidance to strengthen recovery goals and improve subsequent conservation outcomes. In particular, species at risk planners should seek to attain higher recovery goal ambition, particularly for SARA-listed species, and include quantitative recovery goals wherever possible.

## Introduction

Biodiversity is eroding globally as species face unprecedented threats to their existence, including habitat loss and degradation [1,2], exploitation [3], invasive species [4], and climate change [5]. Among the concerted efforts to combat the biodiversity crisis, individual countries and territories often enact national endangered species legislation. Broadly, endangered species legislation aims to identify species at elevated risk of extinction, discern the threats they face, and use this knowledge to protect species and habitats, mitigate species declines, and facilitate species recovery, where feasible. Often far from infallible, national endangered species legislation warrants ongoing scrutiny and constructive change in order to improve the conservation outcomes for the species it is intended to protect.

Canada and the United States (US) share the longest international border in the world and are linked by prominent geographical features such as the Rocky Mountains, the Great Lakes, and countless additional marine, terrestrial, and freshwater ecosystems. In addition to growing lists of species at risk that are unique to each country, Canada and the US also share numerous at-risk species, including migratory (or otherwise mobile) species that traverse both countries and species represented by populations that occur in both countries (e.g., plants and non-migratory species). Two different national legislative acts, Canada’s Species at Risk Act (SARA) and the US’ Endangered Species Act (ESA), aim to identify and protect species at risk in their respective countries.

Identifying and protecting at-risk species in Canada involves multiple steps. Formed in 1977, the Committee on the Status of Endangered Wildlife in Canada (COSEWIC) is an independent scientific body that assesses the status of species potentially at risk of extinction or extirpation within Canada [6]. COSEWIC status assessments, which represent the scientific basis for listing decisions, are subsequently forwarded to the Minister of the Environment [7]. The federal government then either accepts or rejects the Minister’s recommendation, or refers the matter back to COSEWIC for additional information [7]. If accepted, the species is subsequently listed under SARA, which came into force in 2003. Species may be listed as extinct, extirpated, endangered, threatened, or of special concern [7]. A number of protections are then automatically applied to listed species on federal lands, including freshwater and marine systems [7]. SARA’s protections may, upon request by the federal government, be extended to other lands under provincial or territorial jurisdiction [7]. Upon listing, SARA requires Recovery Strategies and Action Plans for endangered and threatened species within specific timeframes [7].

The US federal process for conserving at-risk species and the ecosystems on which they depend was established by ESA legislation enacted in 1973 [8]. Under ESA, species are listed as either endangered or threatened based on their biological status (e.g., population size) and the threats to their continued existence (e.g., habitat destruction, exploitation; [8]). Unlike SARA, species status assessments are conducted by federal agencies (US Fish and Wildlife Service, USFWS; National Marine Fisheries Service, NMFS) and are often initiated by public petitions [9]. Listing determinations are made by these same agencies, accepted or rejected by the Secretary of Commerce (NMFS) or the Secretary of the Interior (USFWS), and legalized when published in the Federal Register. In 1988, amendments to ESA legislation required the development of Recovery Plans for species listed under ESA to describe specific management actions necessary for their recovery [8], the timeframes for which are determined by the responsible federal agency (USFWS or NMFS; [10]).

Previous studies have highlighted the many differences and similarities between ESA and SARA [9,11,12]. Among the key differences, SARA is younger than the ESA and many species protections have yet to be fully implemented [13,14]. Further, while most species at risk in Canada and the US have failed to recover (i.e., the majority of COSEWIC-assessed species at risk: [13]; and most ESA-listed species: [15]), there is evidence that the ESA is effective over time, at least for some species [15]. Importantly, both acts require that Recovery Plans (ESA) and Recovery Strategies (SARA) be produced for species at risk [7,8]); these Recovery Plans and Strategies (hereafter “recovery documents”) outline what is required to assist recovery, recover and/or delist species, including the delineation of objectives for recovering species at risk.

Arguably, and beyond already identified significant issues relating to legislative effectiveness and bias (e.g., SARA: [16–18]; ESA: [19–21]), species recovery planning and subsequent follow-through are crucially important components of national endangered species legislation. Recent publications have drawn attention to variable and, at times, underwhelming ambition of recovery goals under both acts, in addition to a lack of quantitative goals [18,22]. Ambition, defined here as the extent to which recovery goals identify targets that will achieve species recovery, can range from low to high. As an example, ESA recovery goals for the endangered southern resident killer whale (*Orcinus orca*), one of the most intensively studied marine mammals on the planet, are quantitative and of relatively high ambition (“average growth rate of 2.3 percent per year”; [23]). In contrast, recovery goals under SARA are similarly high in ambition but entirely qualitative (“long-term maintenance of […] an increasing size”; [24]). As a second example, although the recovery document contains substantive quantitative population-level information, recovery goals for the northern spotted owl (*Strix occidentalis caurina*) under ESA are qualitative with moderate ambition (“overall population trend […] is stable or increasing”; [25]), whereas goals under SARA are quantitative and more ambitious (“more than 250 mature individuals”; [26]). A third example is the marbled murrelet (*Brachyramphus marmoratus*), another intensively studied species. ESA recovery goals published two decades ago are qualitative but of relatively high ambition (“stabilize and then increase” populations; [27]). Although quantitative, SARA recovery goals are of low ambition, with a short-term (20 year) target being to “halt the decline” and subsequently stabilize the population and nesting habitat areas at less than 30 percent decline compared to 2002 levels [28]. Although it remains unclear whether and how recovery goal ambition is linked to species recovery outcomes, the development of goals with high ambition is likely linked, if not fundamental, to meeting those same goals. In addition, inclusion of quantitative recovery goals offers defined, measurable benchmarks for recovery success (or failure), unlike qualitative goals [29].

Regardless of the drivers (political concerns, socioeconomic considerations, lack of information, and/or an absence of integrated conservation planning; e.g., [12]), varying quantitativeness and/or ambition of recovery goals in Canada and the US raises concerns over conservation effectiveness, efficiency, and ultimately, the persistence of at-risk species. To date, there have been few examinations of the recovery goals of the numerous species listed under endangered species legislation in Canada and the US, including cross-listed species (but see [12]). As multiple delayed recovery documents in Canada [30,31] have been released recently, there is a new opportunity to examine recovery goals for species listed under SARA and ESA.

Initially instigated over the unambiguous contrasts between ESA and SARA recovery goal quality, as measured by quantitativeness and ambition, for several high-profile species (e.g., southern resident killer whale, northern spotted owl, and marbled murrelet), this study’s main objective is to compare and contrast recovery goal quality for species listed under SARA and ESA. Our focus on recovery goal quantitativeness and ambition was strategically identified for the potential to strengthen recovery goal objectives, with the ultimate intent being improved conservation outcomes for species at risk. By comparing across the two legislation types, our aim is to identify differences that could pinpoint areas for improvement as well as similarities that could identify weaknesses common to the development of quality recovery goals under both legislation types. Moreover, our analysis includes a subset of species that are cross-listed under both legislation types, the intent of which is to uncover cases that could benefit from increased cross-border sharing of information. Accordingly, study findings are intended to inform and provide guidance for the improvement of recovery goals in aid of species conservation.

## Methods

We compared the quantitativeness and ambition of recovery objectives for species listed under SARA in Canada and recovery criteria for species listed under the ESA in the United States. We first identified 57 species cross-listed under both SARA and ESA (SARA: http://www.registrelep-sararegistry.gc.ca/sar/index/default_e.cfm; ESA: https://www.fws.gov/endangered/index.html; S1 Table; documents archived 21 August 2019). Recovery documents are required for species listed as endangered or threatened; and endangered, threatened, or extirpated under ESA and SARA, respectively. ESA and SARA cross-listed species were included in our analysis if: (1) they were identified as being the same species or subspecies with a single recovery document under each legislation or; (2) they represented the same population (e.g., white sturgeon, *Acipenser transmontanus*, Kootenay River population) with population-specific recovery documents under both legislation types or; (3) they were equivalent but spatially discrete populations (e.g., beluga whale, *Delphinapterus leucas*), or Designatable Units (DU, Canada; e.g., Sockeye salmon, *Oncorhynchus nerka*) or Distinct Population Segments (DPS, US; e.g., Atlantic salmon, *Salmo salar*) with population-specific recovery documents under both legislation types. Hereafter, we refer to DUs, DPSs, species, subspecies, ecotypes, and subpopulations as “species”. Where available, finalized recovery documents were used; in the absence of a finalized document, draft documents were examined. Two species listed as extirpated in Canada where recovery was deemed either not technically or biologically feasible were included in the summary table (S1 Table) but excluded from subsequent analyses (i.e., prairie population of grizzly bear, *Ursus arctos*; [32]; dwarf wedge mussel, *Alasmidonta heterodon*; [33]). We note that though the black-footed ferret (*Mustela nigripes*) and the karner blue (*Lycaeides melissa samuelis*) are also listed as extirpated in Canada, their recovery is currently considered feasible [34,35]; therefore, these species were retained in our analysis.

Secondly, additional species listed under ESA or SARA were randomly selected to be included in our analysis to supplement the small number of cross-listed species. In total, combining cross-listed and randomly sampled species, we sampled 15 species from each of seven natural taxonomic groupings (i.e., amphibian, bird, fish, invertebrate, mammal, plant, reptile), with exception of SARA-listed amphibians, where there were only 13 recovery documents available (total ESA, n = 105; total SARA, n = 103).

We extracted the recovery goals defined in the recovery documents for cross-listed and randomly selected species and evaluated their quantitativeness and ambition. In ESA recovery documents, recovery goals are defined as “criteria” for reclassifying or delisting, and in SARA documents, these goals are often referred to as “goals” or “objectives” for recovery or, commonly in more recent documents, as “population and distribution objectives”. For ease of interpretation, hereafter, we use the term “recovery goals” to encompass the terms and criteria described above. To directly compare recovery goals, we scored each recovery document according to two attributes:

1. Quantitativeness of recovery goals: we first assessed whether the documents included quantitative or qualitative recovery goals. Goals were considered quantitative if a numeric target (e.g., population size, number of breeding pairs, percent population increase) was included. For plants, we also considered area of occupancy or extent occurrence as numeric population targets due to the difficulty of estimating number of individuals for some species. We scored only the recovery goal, and not the methods used to assess a species status or generate population trajectories within the entire recovery document. Some species’ recovery documents were quantitative throughout their methods sections (e.g., demographic analyses), but lacked a quantitative element in their recovery goal; these recovery goals were scored as qualitative. For example, despite extensive quantitative analyses in other sections of the ESA recovery plan for the northern spotted owl, the recovery goal of “overall population trend […] is stable or increasing” [25] was scored as a qualitative goal. Where no goal was included, this attribute was assigned “NA”.
2. Ambition of recovery goals: recovery goals were assigned an ambition score modified from McCune et al. [18]. Ambition, defined as the extent to which recovery goals delineate targets that will achieve species recovery, was described using a five-point scale that ranged from low to high (Table 1).

**Table 1:** Recovery ambition scoring guide for species’ recovery plans and strategies under Canada’s Species at Risk Act (SARA) and the US Endangered Species Act (ESA), modified from McCune et al. [18].

Ambition score	Recovery goal	Quantitative example	Qualitative example	Species with matching ambition score where one legislation is quantitative and the other qualitative
1	None stated (e.g. goal related to protection of area but not regarding population size)	SARA: none	SARA: Kirkland’s warbler (*Setophaga kirtlandii/Dendroica kirtlandii*)	None
		ESA: none	ESA: western prairie fringed orchid (*Platanthera praeclara)*	
2	Vague (e.g., “maintain an index of area of occupancy” [36], “achieve sufficient and viable populations” [37]—without clarification as to what this means) or less than current level	SARA: marbled murrelet (*Brachyramphus marmoratus*)	SARA: northern riffleshell (*Epioblasma torulosa rangiana*)	None
		ESA: sei whale (*Balaenoptera borealis)*	ESA: dwarf lake iris (*Iris lacustris*)	
3	Equal to current level (e.g., population is stable), or willing to accept current level or increase (e.g. “population is stable or increasing”[38], or “maintain or increase” [39])	SARA: none	SARA: short-tailed albatross (*Phoebastria albatrus*)	Species: Spalding’s catchfly (*Silene spaldingii*)
		ESA: whooping crane (*Grus Americana*)	ESA: northern spotted owl (*Strix occidentalis caurina*)	Quantitative: ESA
				Qualitative: SARA
4	Restore to levels greater than current, restore to levels greater than current but less than historic, or restore to levels greater than current with historic levels unknown	SARA: northern spotted owl (*Strix occidentalis caurina*)	SARA: north Atlantic right whale (*Eubalaena glacialis*)	Species: golden paintbrush (*Castilleja levisecta*)
		ESA: short-tailed albatross (*Phoebastria albatrus)*	ESA: marbled murrelet (*Brachyramphus marmoratus*)	Quantitative: ESA
				Qualitative: SARA
5	Restore to historic levels; however, must include description of historic population extent and time period	SARA: none	SARA: none	None
		ESA: none	ESA: none	

Additionally, recovery goals in SARA recovery documents were often separated into short- and long-term objectives; typically these two time periods were based on the life history of the species in question, with several exceptions where time frames were not clarified (in these cases, objectives were categorized as long-term). When present, the two time frames were assessed separately. The two time frames often received different scores for both recovery goal quantitativeness and ambition and thus, as a conservative approach for the direct comparison between SARA and ESA recovery documents, we used the “best case scenario” from the two SARA goals by choosing quantitative over qualitative goals and using the higher ambition score. Once completed, 10% of recovery documents were randomly re-sampled and evaluated again by three trained individuals; on average, repeatability scoring for recovery goal ambition score and recovery goal quantitativeness was 90%.

### Data analysis

We used Fisher’s Exact tests to examine similarities and differences in quantitativeness and ambition in relation to legislation type, species status, and natural taxonomic grouping. For simplicity, we compared goals with ambition scores of 4 (i.e., the highest-ranked goals in the data set) to all lower ranked goals (i.e., 1, 2, or 3). For comparisons by status, we focused on species listed as threatened versus endangered due to the small number of species in our dataset (n = 2) listed as extirpated under SARA. All data were analyzed and graphed using R statistical software [40].

## Results

Considering all recovery documents examined in this study, 38% (78/208) of recovery goals were quantitative and 41% (85/208) had high ambition (i.e., an ambition score of 4). Only 26% (55/208) of recovery goals were both quantitative and had high ambition. Overall, quantitative goals were associated with higher ambition (Table 2). Specifically, the odds of a quantitative goal receiving an ambition score of 4 were 7.9 times higher than for a qualitative goal. The trend of quantitative goals having higher ambition was consistent in both legislation types (Table 2). However, and relevant to evaluating the potential redundancy of the scoring system applied in this study, the two metrics of recovery goal quality (i.e., quantitativeness and an ambition score of 4) differed for 25% (53/208) of goals evaluated. In other words, 25% of goals were scored as either quantitative with low ambition (23/208) or qualitative with high ambition (30/208).

**Table 2:** Comparisons of the quantitativeness and ambition of recovery goals (n = 208) by legislation, status, natural grouping, and habitat. Odds ratios and confidence intervals were obtained from Fisher’s Exact Tests. See Methods for further detail. Cells shaded grey with italicized text denotes odds ratios with confidence intervals that overlap one (i.e., include the possibility of no difference).

Comparison		Proportion of Goals (%)		Odds Ratio (95% CI)
**Quantitativeness**		**Quant**	**Qual**	
**of Ambition 4**	Overall	55/78 (71)	30/130 (23)	7.9 (4.0–15.8)
**Goals**	ESA	36/51 (71)	17/54 (31)	5.1 (2.1–13.1)
SARA		19/27 (70)	13/76 (17)	11.1 (3.7–36.6)
**Legislation**		**ESA**	**SARA**	
Quantitative		51/105 (49)	27/103 (26)	2.6 (1.4–5.0)
Ambition 4		53/105 (50)	32/103 (31)	2.4 (1.3–4.4)
**Status**		**Endangered**	**Threatened**	
Quantitative	Overall	62/147 (42)	15/59 (25)	2.1 (1.1–4.5)
ESA		42/77 (54)	9/28 (32)	*2*.*5 (0*.*9–7*.*2)*
SARA		20/70 (29)	6/25 (19)	*1*.*7 (0*.*6–5*.*7)*
Ambition 4	Overall	68/147 (46)	17/59 (29)	2.1 (1.1–4.4)
ESA		42/77 (54)	11/28 (39)	*1*.*8 (0*.*7–5*.*0)*
SARA		26/70 (37)	6/31 (19)	*2*.*4 (0*.*8–8*.*3)*
**Natural Grouping**		**ESA**	**SARA**	
Quantitative	Mammals	11/15 (73)	5/15 (33)	*5*.*2 (0*.*9–35*.*6)*
Birds		12/15 (80)	10/15 (67)	*1*.*9 (0*.*3–15*.*8)*
Reptiles		6/15 (40)	1/15 (7)	*8*.*7 (0*.*8–457*.*9)*
Amphibians		5/15 (33)	1/13 (8)	*5*.*6 (0*.*5–306*.*3)*
Fish		6/15 (40)	6/15 (40)	*1 (0*.*2–5*.*5)*
Invertebrates		1/15 (7)	2/15 (13)	*0*.*5 (0–10*.*2)*
Plants		10/15 (67)	2/15 (13)	11.7 (1.7–147.7)
Ambition 4	Mammals	11/15 (73)	6/15 (40)	*3*.*9 (07–25*.*9)*
Birds		8/15 (53)	7/15 (47)	*1*.*2 (0*.*2–6*.*9)*
Reptiles		7/15 (47)	0/15 (0)	*Infinity*
Amphibians		7/15 (47)	2/15 (13)	*4*.*5 (0*.*6–56*.*3)*
Fish		7/15 (47)	10/15 (67)	*0*.*5 (0*.*1–2*.*4)*
Invertebrates		6/15 (40)	3/15 (20)	*2*.*6 (0*.*4–20*.*4)*
Plants		7/15 (47)	4/15 (27)	*2*.*3 (0*.*4–15*.*0)*

**Fig 1:**
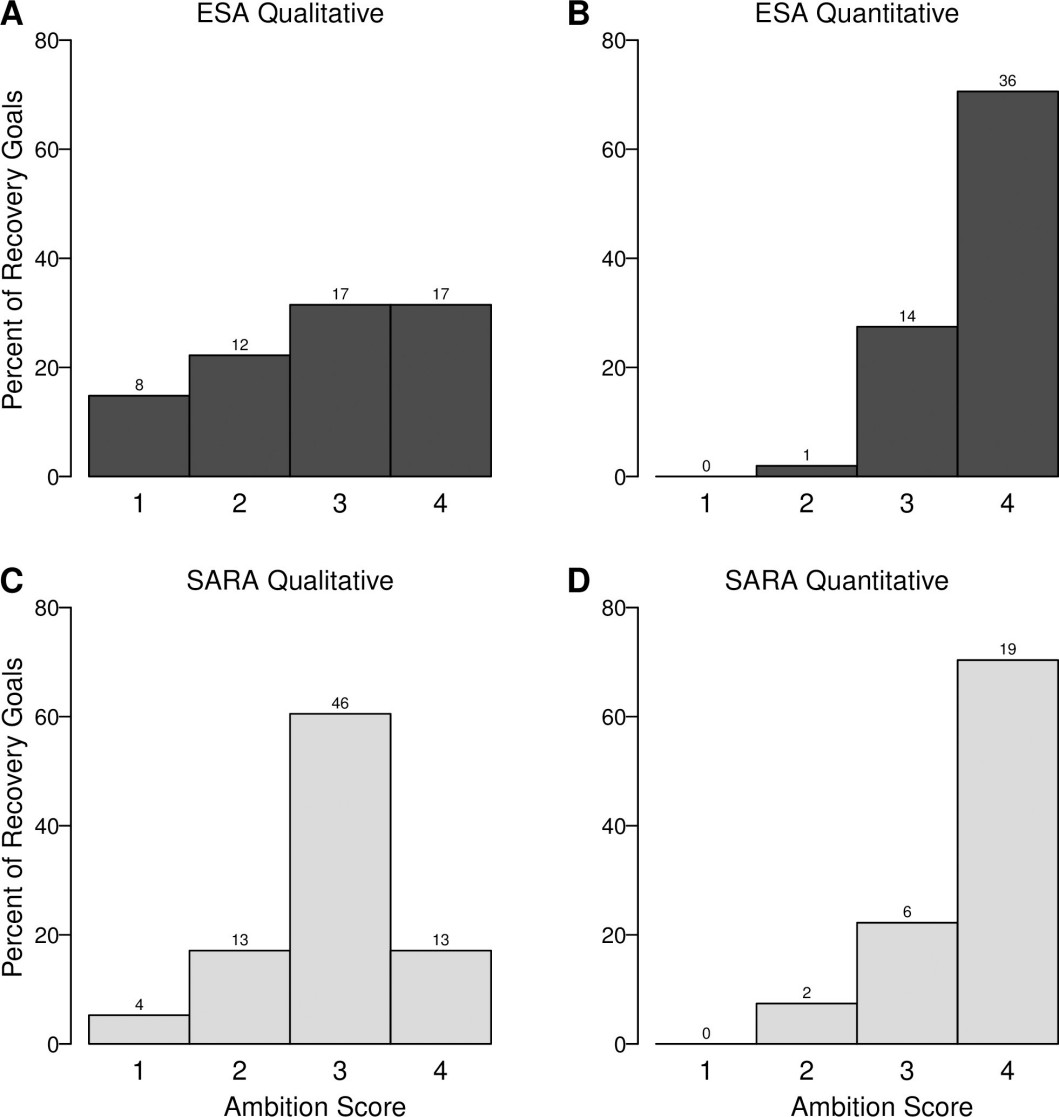
Ambition of qualitative and quantitative recovery goals for species (n = 208) listed under the United States’ Endangered Species Act (ESA) and Canada’s Species at Risk Act (SARA). Bars show the percent of recovery goals with ambition scores 1–4 for (A) qualitative goals under the ESA, (B) quantitative goals under the ESA, (C) qualitative goals under SARA, and (D) quantitative goals under SARA. Ambition of recovery goals within published recovery documents was scored on a scale from 1 to 5; however, no goals qualified for an ambition score of 5 (see Methods). The number of recovery goals in each category is displayed above each bar.

Comparing between legislation types, ESA recovery goals were more quantitative and had higher ambition than SARA recovery goals (Fig 1; Table 2). Specifically, the odds of a quantitative goal were 2.6 times higher under ESA than SARA (Table 2). Similarly, the odds of a goal receiving an ambition score of 4 were 2.4 times higher under ESA than SARA (Table 2). The trend of ESA goals having higher ambition than SARA goals occurred primarily among qualitative goals, where the mode ambition scores were 4 and 3 for ESA and SARA, respectively (Fig 1A and 1C). By contrast, the proportion of quantitative goals with an ambition score of 4 were similar for both ESA and SARA (Fig 1B and 1D). Notably, no recovery goals scored an ambition score of 5 (Fig 1).

**Fig 2:**
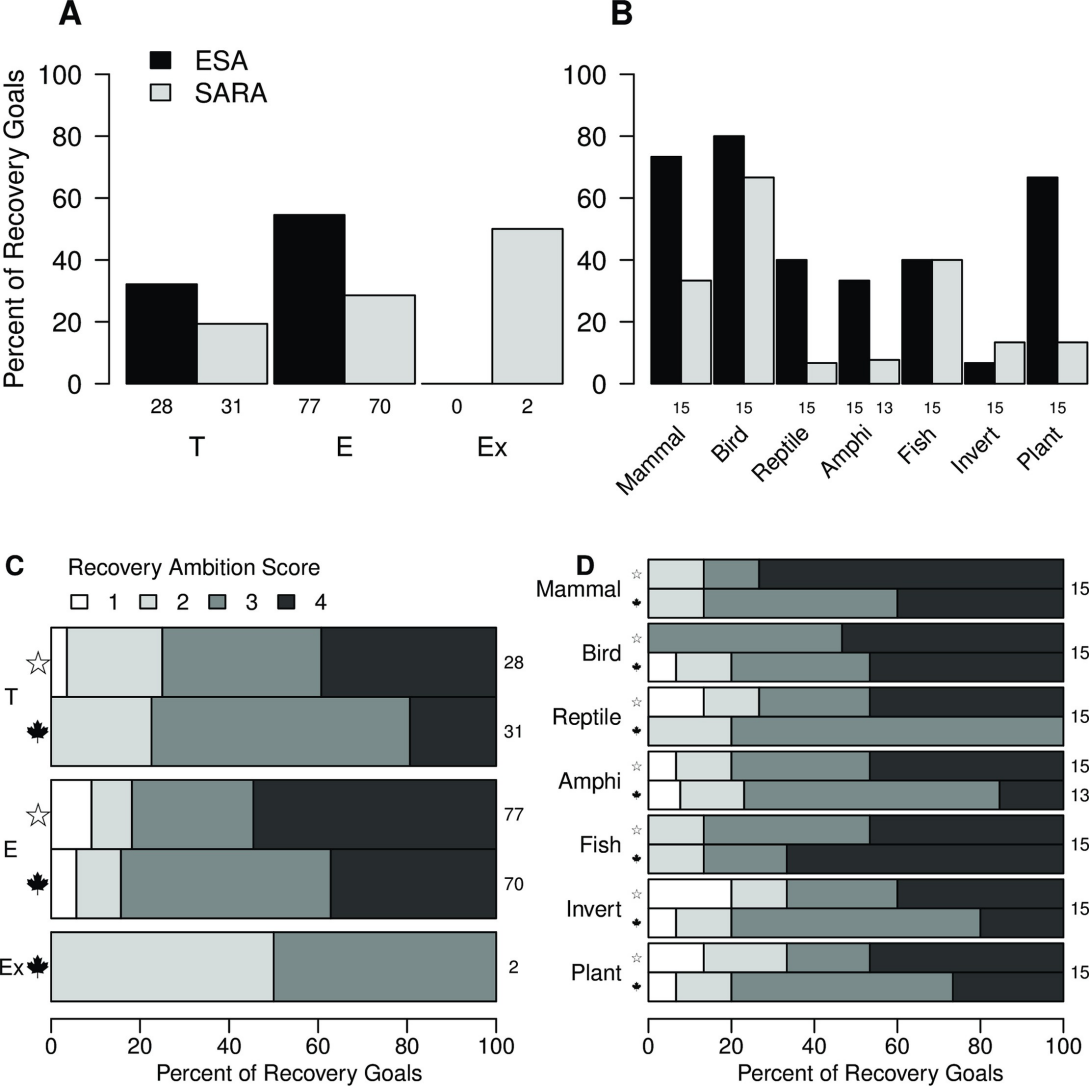
Quantitativeness and ambition of recovery goals for species (n = 208) listed under both the United States’ Endangered Species Act (ESA) and Canada’s Species at Risk Act. Percent of quantitative recovery goals (A) by species status (i.e., threatened [T], endangered [E], or extirpated [Ex]), and (B) by natural grouping. Ambition of recovery goals listed under the ESA (denoted by a star) and SARA (denoted by a maple leaf) (C) by status and (D) natural grouping, Ambition of recovery goals within published recovery documents was scored on a scale from 1 to 5; however, no goals received an ambition score of 5 (see Methods). Sample sizes are displayed to the right of bars.

Quantitativeness and ambition also differed by species status (Table 2). Overall, the odds of a goal being quantitative were 2.1 times higher for endangered than for threatened species (Fig 2A; Table 2). Similarly, the odds of a goal receiving an ambition score of 4 were 2.1 times higher for endangered than for threatened species (Fig 2C; Table 2). When considering each legislation type separately, the trend of goals for endangered species being more quantitative and ambitious than those for threatened species were consistent; however, the confidence intervals of the odds ratios for each legislation type overlapped one (i.e., included the possibility of no difference by status; Table 2; Fig 2A and 2C).

The trend of ESA being more quantitative and having higher ambition than SARA was largely consistent across natural taxonomic groupings (Fig 2B and 2D; Table 2). One exception was in fish, where SARA goals had a similar proportion of quantitative goals and a higher proportion of goals with an ambition score of 4 compared with ESA goals (Fig 2B and 2D). A second exception was for invertebrates, where the proportion of quantitative goals was low for both legislation types (Fig 2B). Comparing across natural groupings, the greatest difference in quantitativeness between legislation types occurred in plants, where the odds of a quantitative goal were 11.7 times higher under ESA than SARA (Fig 2B; Table 2). The difference in recovery goal ambition was greatest in reptiles; however, it was not possible to calculate an odds ratio because no SARA goals for reptiles had an ambition score of 4 (Fig 2D; Table 2).

**Fig 3:**
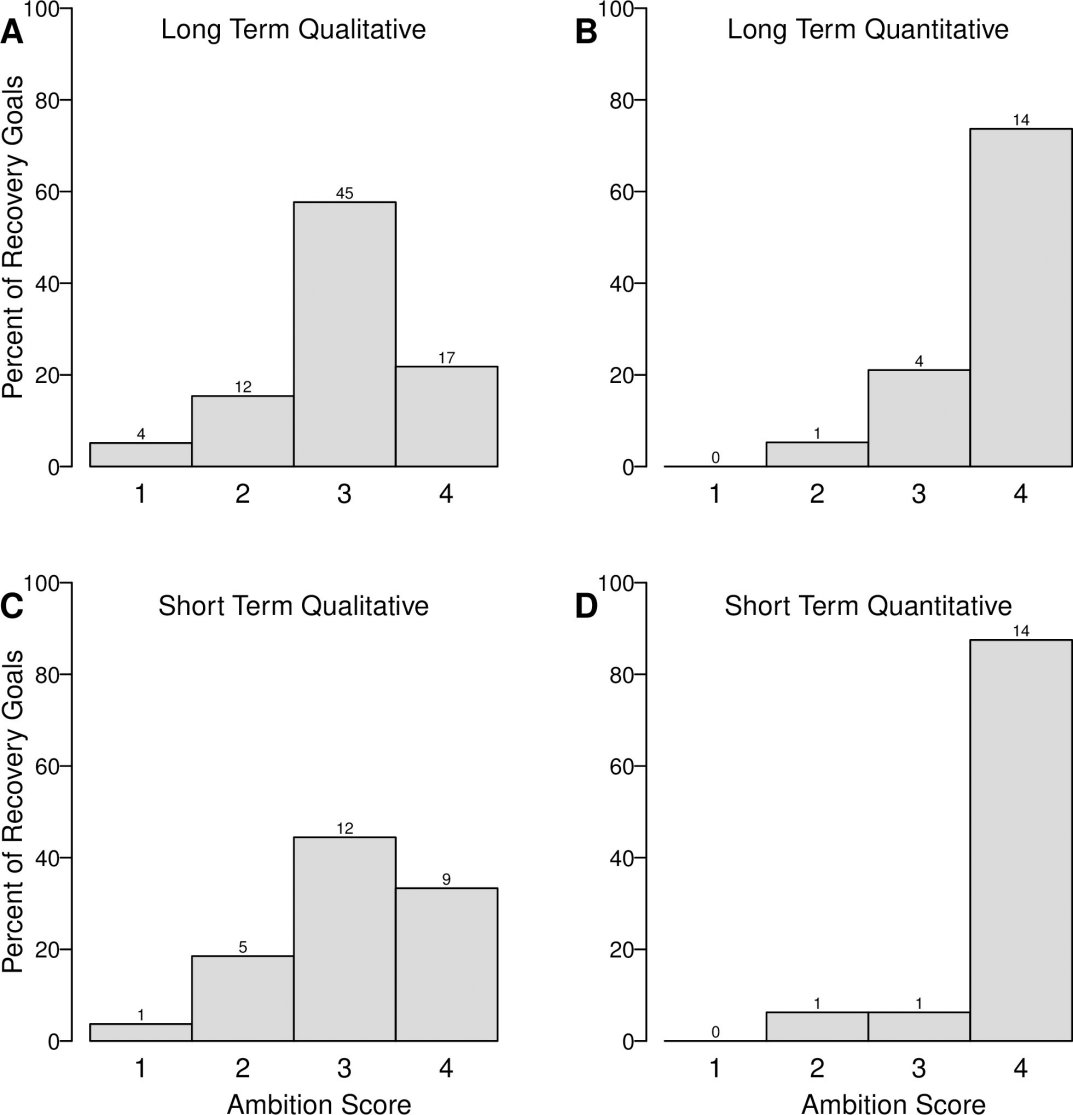
Ambition of qualitative and quantitative long-term (n = 97) and short-term (n = 43) recovery goals for species listed under Canada’s Species at Risk Act (SARA). Bars show the percent of recovery goals with ambition scores 1–4 for (A) long-term qualitative goals, (B) long-term quantitative goals, (C) short-term qualitative goals, and (D) short-term quantitative goals. Ambition of recovery goals within published recovery documents was scored on a scale from 1 to 5; however, no goals received an ambition score of 5 (see Methods). The number of goals in each category is displayed above each bar. Sample sizes are uneven because seven recovery plans contained only long-term goals and one strategy contained only short-term goals.

The majority of long- and short-term recovery goals for SARA-listed species were qualitative ($\frac{78}{97}=80\%$ and $\frac{27}{43}=63\%$ respectively; Fig 3). In both the long-term and short-term, quantitative goals had higher ambition (mode = 4; Fig 3B and 3D) than qualitative goals (mode = 3; Fig 3A and 3C).

The majority of species cross-listed under SARA and ESA had recovery documents ($\frac{36}{57}=63\%$, including three extirpated species in Canada; S1 Table). Twelve $\left(\frac{12}{57}=21\%\right)$ cross-listed species had only one recovery document, either by SARA or ESA. Only two species are currently without a recovery document from either country ($\frac{2}{57}=4\%$), one of which is the Vancouver Island marmot, which is considered a Foreign Species under ESA. Lastly, seven species ($\frac{7}{57}=12\%$) were listed as Special Concern under SARA (where no Recovery Strategy is required) and as endangered or threatened under ESA (four of which had Recovery Plans).

Our study findings for the subset of species listed under both legislation types (n = 34; S2 Table; S1–S3 Figs) were largely consistent with findings from the full complement of recovery documents examined in this study (n = 208; Table 2). An exception is that the trend of endangered species having a higher proportion of quantitative goals compared with threatened species is consistent for both legislation types in the larger data set, whereas the trend is reversed for species listed under SARA in the cross-listed data set (S2 Table). Relevant to potential information-sharing between countries, 18 of 34 species (53%) listed under both legislation types had goals that were quantitative under one legislation but not the other, including 13 cases where ESA goals were quantitative but SARA goals were not and 5 cases where SARA goals were quantitative and ESA goals were not. Similarly, 18 of 34 the cross-listed species (53%) had high ambition (ambition score of 4) under one legislation but not the other, including 14 cases where ESA goals had high ambition but SARA goals did not and 4 cases where SARA goals had high ambition but ESA goals did not.

## Discussion

Endangered species legislation is essential to protect and recover at-risk species, but its successful implementation is inherently difficult, requiring considerable time, cost, and species-specific information. Previous assessments of national endangered species legislation and associated processes have identified multiple areas for improvement (e.g., listing process bias, critical habitat designation, and delays; [21,30]); recovery documents also offer a potentially rapid, strategic avenue for improving species’ conservation status [e.g., [29]. Although reviews of recovery documents provide guidance on threat monitoring [20], the biological relevance of recovery criteria [41], and recovery document scope [42], this study focused on the quality (i.e., quantitativeness and ambition) of recovery goals as a key component of effective recovery documents. Overall, 38% of goals were quantitative, 41% had high ambition (i.e., an ambition score of 4), and 26% were both quantitative and highly ambitious. Comparing between legislation types, ESA recovery goals generally were more quantitative and had higher ambition than those put forward under SARA. Common to both legislations, recovery goals for endangered species were more quantitative and had higher ambition than those for threatened species. By focusing on listed species with recovery documents, our study provides an appraisal of recovery goal quality for species at risk, and is intended to contribute to efforts to strengthen recovery goals and subsequent conservation outcomes for species in Canada and the US.

One of our main findings was that only 38% of recovery documents assessed had recovery goals that were quantitative. This finding is highly relevant, as recovery goal quality may affect conservation outcomes. As a key example, Gerber and Hatch [29] found that quantitative recovery goals were associated with improved status of ESA-listed species. Further, quantitative recovery goals have been praised for providing consistency, efficiency, transparency, and legitimacy; consequently, recent reviews have called for their inclusion in recovery documents whenever possible [43]. Despite these strengths, quantitative conservation targets have been criticized for their rigidity that may not be adaptive to complex ecological variation (e.g., climate change response) or socioeconomic criteria [44], for their application in situations when they are not biologically founded [45,46], and in cases where inherent uncertainty around targets is not reported [47]. When these concerns can be addressed, our results indicate that recovery goal quantitativeness should be improved where possible.

Regarding ambition, only 41% of goals had high ambition (i.e., received an ambition score of 4). Although recovery goals with higher ambition were more likely to be quantitative, the two measures of goal quality differed (i.e., were scored as either quantitative with low ambition or qualitative with high ambition) for 25% of species examined (see Table 1 for examples). These differences suggest that both measures of recovery goal quality should be considered when developing recovery goal targets. An important next step, however, would be to explore the relationships between recovery goal ambition and species’ conservation status outcomes under SARA and ESA.

Contributing new knowledge regarding the differences and similarities between ESA and SARA, this study also finds that SARA recovery goals are generally less quantitative and of lower ambition than those put forward under ESA. While recovery goal quality could be improved under both legislative acts, this appears to be particularly the case for SARA-listed species in Canada. Further, for the subset of species cross-listed under both ESA and SARA, there is evidence of varying goal quality between the two legislations, with SARA-listed species having lower recovery goal quality in general, in terms of both ambition and quantitativeness. Recovery goals with underwhelming ambition (i.e., ambition score 1 or 2) have been previously identified for SARA-listed species [17], but direct comparison with ESA species and the subset of cross-listed species has not been previously undertaken. Whereas McCune et al. [17] speculated that a species’ economic importance may influence recovery goal ambition, other factors may also contribute to reduced recovery goal quality under SARA.

Although there are a litany of potential drivers for the observed differences in recovery goal quality between ESA and SARA, several fundamental explanations represent starting points for further study. First, ESA is older than SARA and has been subject to amendments, arguably greater academic scrutiny [19,42,48], and more legal challenges (e.g., Centre for Biological Diversity; http://www.biologicaldiversity.org) as compared to SARA (but see [17,18]). Funding also differs greatly between the two legislations; for example, in 2007, the average amount spent per species was 1.3 million USD under ESA versus 0.2 million USD under SARA [49,50]. Although little explored, funding disparities could potentially influence any stage of endangered legislation implementation, including the setting and achievement of recovery goals. For example, Miller et al. [51] found that species listed under the ESA with higher funding were more likely to have either stable or increasing status. Institutional perspectives and language typically used in the two recovery document types also frequently differed; these differences may have affected recovery goal quality. As an example, ESA recovery documents tended to use more explicit language (e.g., “stable populations or increasing”; [27]) compared with often vague language used in SARA recovery documents (e.g., “maintain long-term, self-sustaining, viable populations”; [52]). Finally, fundamental differences in the requirements laid out by the two acts may also influence recovery goal quality [9]; as an example, recent ESA recovery documents are required to include criteria necessary for delisting [8], unlike SARA.

As a third major finding, recovery goal quantitativeness and ambition were higher for species listed as endangered as compared to threatened under ESA and SARA. Higher recovery goal quality for endangered species could be related to a greater urgency to increase endangered populations rather than maintain or slow their decline [53]. Alternatively or concomitantly, the difference could relate to better knowledge of endangered species, leading to quantitative, high ambition recovery goals for endangered species [43,54]. Regardless of the drivers, differences in recovery goal quality for endangered and threatened species have implications for the timing of recovery interventions in a species’ pathway to recovery, including a failure to recover. Although allocating resources to the most endangered species can have benefits in the short term, investments in less endangered species benefit more species in the longer term [55]. Further examination of these differences, and potential ramifications for conservation outcomes, remain to be explored.

In the midst of a global extinction crisis, effective conservation actions for recovering species at risk are crucial. When setting targets for at-risk species recovery, low quality recovery goals may undermine or stymie conservation efforts and, thus, are counterproductive. Given the potentially negative conservation outcomes associated with low quality goals, [e.g., [19], our results provide clear guidance for developing higher quality goals for recovery planning. Specifically, our findings highlight that recovery goals could be improved by focusing on the development of unambiguous, high-ambition (i.e., increasing population), and quantitative recovery goals wherever possible. Further, differences in recovery goal quality between ESA and SARA indicate that greater international information sharing [11] could provide a synergistic benefit in achieving conservation outcomes (e.g., via data and expertise sharing, joint recovery planning, and cross-border species management) for species cross-listed under both legislative acts. Ultimately, increasing recovery goal ambition for species with lower threat status could result in stronger conservation outcomes, although this topic warrants additional exploration. As a final point, attention to recovery goal quality is important because recovery documents are accessed by diverse users, including those without a scientific background and/or species-specific knowledge; by extension, recovery goals should be straightforward, explicit, and standalone. Although many other aspects of endangered species legislation require improvement, increasing recovery goal quality represents a strategic opportunity to effect meaningful and urgently needed conservation outcomes.

## Supporting information

S1 Table Identity and status of cross-listed species at risk under Canada's Species at Risk Act (SARA) and the US Endangered Species Act (ESA). Recovery documents not finalized as of 21 August 2019 are denoted with “NF”. “Pop(s)” refers to population(s) identified by the recovery documents.
(DOCX)

S2 Table Comparisons of the quantitativeness and ambition of recovery goals by legislation, status, natural grouping, and habitat for species listed under both SARA and ESA (n = 34). Odds ratios and confidence intervals were obtained from Fisher’s Exact Tests. See Methods for further detail. Shading shows where the trend was opposite between the subset of paired data (n = 34) and the larger set of recovery documents (n = 208).
(DOCX)

S1 Fig Ambition of qualitative and quantitative recovery goals for species (n = 34) listed under the United States’ Endangered Species Act (ESA) and Canada’s Species at Risk Act (SARA). Bars show the percentage of recovery goals with ambition scores 1–4 for (A) qualitative goals under the ESA, (B) quantitative goals under the ESA, (C) qualitative goals under SARA, and (D) quantitative goals under SARA. Ambition of recovery goals within published recovery documents was scored on a scale from 1 to 5; however, no goals qualified for an ambition score of 5 (see Methods). The number of goals in each category is displayed above each bar.
(TIF)

S2 Fig Quantitativeness and ambition of recovery goals for species (n = 34) listed under both the United States’ Endangered Species Act (ESA) and Canada’s Species at Risk Act. Percent of quantitative recovery goals (A) by species status (i.e., threatened [T], endangered [E], or extirpated [Ex]), and (B) by natural grouping. Ambition of recovery goals listed under the ESA (denoted by a star) and SARA (denoted by a maple leaf) (C) by status and (D) natural grouping. Ambition of recovery goals within published recovery documents was scored on a scale from 1 to 5; however, no goals received an ambition score of 5 (see Methods). Sample sizes are displayed to the right of bars.
(TIF)

S3 Fig Ambition of qualitative and quantitative long-term and short-term recovery goals for species (n = 34) listed under Canada’s Species at Risk Act (SARA). Bars show the percent of recovery goals with ambition scores 1–4 for (A) long-term qualitative goals, (B) long-term quantitative goals, (C) short term qualitative goals, and (D) short-term quantitative goals. Ambition of recovery goals within published recovery documents was scored on a scale from 1 to 5; however, no goals received an ambition score of 5 (see Methods). The number of goals in each category is displayed above each bar. Only species cross-listed under the United States’ Endangered Species Act are shown. Sample sizes are uneven because seven recovery plans contained only long-term goals and one strategy contained only short-term goals.
(TIF)
