# Supplementary material for: Raising the bar: Recovery ambition for species at risk in Canada and the US
Source: PLoS One. 2019 Nov 19;14(11):e0224021. doi: 10.1371/journal.pone.0224021 (PMC6863564; doi:10.1371/journal.pone.0224021)
Supplement: S1 Table — Recovery documents not finalized as of 21 August 2019 are denoted with “NF”. “Pop(s)” refers to population(s) identified by the recovery documents. (DOCX) [file pone.0224021.s001.docx]

**S1 Table. Identity and status of cross-listed species at risk under Canada's Species at Risk Act (SARA) and the US Endangered Species Act (ESA).**

| **Scientific Name** | **Common Name** | **Pop(s)** | **Natural Group** | **ESA** | | | **SARA** | | |
| --- | --- | --- | --- | --- | --- | --- | --- | --- | --- |
|  |  |  |  | **Status** | **Year Listed** | **Plan Finalized** | **Status** | **Year Listed** | **Strategy Finalized** |
| Species with Recovery Plans (ESA) and Recovery Strategies (SARA) | | | | | | | | | |
| *Strix occidentalis caurina* | Northern spotted owl | Entire (ESA), BC (SARA) | bird | T | 1990 | 2011 | E | 2003 | 2006 |
| *Sterna dougallii* | Roseate tern | Northeastern Population (ESA), Entire (SARA) | bird | E | 1987 | 1998 | E | 2003 | 2010 |
| *Charadrius melodus melodus* | Piping plover | Atlantic Coast (ESA), *Charadrius melodus melodus* (SARA) | bird | T | 1986 | 1996 | E | 2003 | 2012 |
| *Charadrius melodus circumcinctus* | Piping plover | Great lakes (ESA), *Charadrius melodus circumcinctus* (SARA) | bird | E | 1985 | 2003 | E | 2003 | 2006 |
| *Grus americana* | Whooping crane |  | bird | E | 1967 | 2007 | E | 2003 | 2007 |
| *Setophaga kirtlandii/Dendroica kirtlandii* | Kirtland's warbler |  | bird | E | 1967 | 1985 | E | 2003 | 2006 |
| *Acipenser transmontanus* | White sturgeon | Kootenai/Kootenay River (ESA & SARA) | fish | E | 1994 | 1999 | E | 2006 | 2014 |
| *Salmo salar* | Atlantic salmon | Gulf of Maine (ESA), Inner Bay of Fundy (SARA) | fish | E | 2000 | 2005 | E | 2003 | 2010 |
| *Balaenoptera borealis* | Sei whale | Entire (ESA), Pacific (SARA) | mammal | E | 1970 | 2011 | E | 2005 | 2006 |
| *Eubalaena glacialis* | North Atlantic right whale |  | mammal | E | 1970 | 2005 | E | 2004 | 2014 |
| *Eubalaena japonica* | North Pacific right whale | North Pacific Ocean and North Atlantic Ocean (ESA), Northeast Pacific (SARA) | mammal | E | 1970 | 2013 | E | 2006 | 2011 |
| *Balaenoptera musculus* | Blue whale | North Atlantic and North Pacific Populations (ESA) and Northwest Atlantic (SARA) | mammal | E | 1970 | 1998 | E | 2005 | 2009 |
| *Orcinus orca* | Killer whale | Southern Resident (ESA & SARA) | mammal | E | 2006 | 2008 | E | 2003 | 2011 |
| *Rangifer tarandus caribou* | Woodland caribou | Selkirk Mountain Population (ESA), Southern Mountain Population (SARA) | mammal | E | 1983 | 1994 | E | 2003 | 2014 |
| *Epioblasma torulosa rangiana* | Northern riffleshell |  | invertebrate | E | 1993 | 1994 | E | 2003 | 2007 |
| *Castilleja levisecta* | Golden paintbrush |  | plant | T | 1997 | 2000 | E | 2003 | 2006 |
| *Cirsium pitcheri* | Pitcher's thistle |  | plant | T | 1988 | 2002 | E | 2003 | 2011 |
| *Isotria medeoloides* | Small whorled pogonia |  | plant | T | 1982 | 1992 | E | 2003 | 2007 |
| *Pedicularis furbishiae* | Furbish's lousewort |  | plant | E | 1978 | 1991 | E | 2003 | 2010 |
| *Platanthera leucophaea* | Eastern prairie fringed orchid |  | plant | T | 1989 | 1999 | E | 2005 | 2012 |
| *Platanthera praeclara* | Western prairie fringed orchid |  | plant | T | 1989 | 1996 | E | 2003 | 2006 |
| *Silene spaldingii* | Spalding's catchfly (Spalding's campion) |  | plant | T | 2001 | 2008 | E | 2005 | 2017 |
| *Dermochelys coriacea* | Leatherback sea turtle | Pacific (ESA & SARA) | reptile | E | 1970 | 1998 | E | 2003 | 2006 |
| *Dermochelys coriacea* | Leatherback sea turtle | Atlantic (ESA & SARA) | reptile | E | 1970 | 1992 | E | 2003 | 2006 |
| *Mustela nigripes^1^* | Black-footed ferret |  | mammal | E | 1967 | 2013 | Ex | 2003 | 2009 |
| *Ursus arctos^2^* | Grizzly bear | Entire (ESA), Prairie population (SARA) | mammal | T | 1975 | 1993 | Ex | 2003 | 2009 |
| *Alasmidonta heterodon^2^* | Dwarf wedgemussel |  | invertebrate | E | 1990 | 1993 | Ex | 2003 | 2007 |
| *Brachyramphus marmoratus* | Marbled murrelet | Entire (BC only, SARA), Washington, Oregon, California (ESA) | bird | T | 1992 | 1997 | T | 2003 | 2014 |
| *Phoebastria albatrus* | Short-tailed albatross |  | bird | E | 1970 | 2008 | T | 2005 | 2008 |
| *Balaenoptera musculus* | Blue whale | North Atlantic and North Pacific Populations (ESA), Pacific (SARA) | mammal | E | 1970 | 1998 | T | 2005 | 2006 |
| *Balaenoptera physalus* | Fin whale | Entire (ESA), Pacific (SARA) | mammal | E | 1970 | 2010 | T | 2006 | 2009 |
| *Megaptera novaeangliae^3^* | Humpback whale | North Atlantic and North Pacific (ESA), North Pacific (SARA) | mammal | E | 1970 | 1991 | T | 2005 | 2013 |
| *Tetraneuris herbacea* | Lakeside daisy |  | plant | T | 1988 | 1990 | T | 2004 | 2011 |
| *Iris lacustris* | Dwarf lake iris |  | plant | T | 1988 | 2013 | T | 2006 | 2011 |
| *Rhinichthys osculus* | Foskett speckled dace |  | fish | T | 1985 | 1998 | E | 2009 | 2018 |
| *Lycaeides melissa samuelis* | Karner blue |  | invertebrate | E | 1992 | 2003 | Ex | 2003 | 2019 |
| Species with Recovery Plans (ESA) or Recovery Strategies (SARA) only | | | | | | | | | |
| *Calidris canutus rufa* | Red knot (*rufa* subspecies) |  | bird | T | 2015 | - | E | 2012 | 2017 |
| *Epioblasma triquetra* | Snuffbox mussel |  | invertebrate | E | 2012 | - | E | 2003 | 2006 |
| *Eremophila alpestris strigata* | Horned lark (*strigata* subspecies) |  | bird | T | 2013 | - | E | 2005 | 2007 |
| *Euphydryas editha taylori* | Taylor's checkerspot |  | invertebrate | E | 2013 | - | E | 2003 | 2006 |
| *Myotis septentrionalis* | Northern myotis |  | mammal | T | 2015 | - | E | 2014 | 2018 |
| *Numenius borealis^4^* | Eskimo curlew |  | bird | E | 1973 | - | E | 2003 | 2007 |
| *Rana pretiosa* | Oregon spotted frog |  | amphibian | T | 2014 | - | E | 2003 | 2015 |
| *Villosa fabalis* | Rayed bean |  | invertebrate | E | 2012 | - | E | 2003 | 2006 |
| *Delphinapterus leucas* | Beluga whale | Cook Inlet (ESA), St Lawrence (SARA) | mammal | E | 2011 | - | T | 2005 | 2012 |
| *Hesperia dacotae* | Dakota skipper |  | invertebrate | T | 2014 | - | T | 2005 | 2007 |
| *Oarisma poweshiek* | Poweshiek skipperling |  | invertebrate | E | 2014 | - | T | 2005 | 2012 |
| Species without Recovery Plans (ESA) or Recovery Strategies (SARA) | | | | | | | | | |
| *Marmota vancouverensis*^5^ | Vancouver Island marmot |  | mammal | E | 1984 | - | E | 2003 | NF |
| *Accipiter gentilis laingi* | Northern goshawk (*laingi* subspecies) |  | bird | T | 2012 | - | T | 2004 | 2018 |
| *Bison bison athabascae* | Wood bison |  | mammal | T | 1973 | - | T | 2003 | 2018 |
| Species Listed as Special Concern (SARA) | | | | | | | | | |
| *Acipenser brevirostrum* | Shortnose sturgeon |  | fish | E | 1967 | 1998 | SC | 2009 | - |
| *Acipenser medirostris* | Green sturgeon |  | fish | T | 2006 | - | SC | 2006 | - |
| *Balaena mysticetus* | Bowhead whale | Alaska (ESA), Bering-Chukchi-Beaufort (SARA) | mammal | E | 1970 | - | SC | 2003 | - |
| *Eumetopias jubatus* | Steller sea lion | Eastern and Western Distinct Population Segments (ESA) | mammal | E | 1997 | 2008 | SC | 2005 | - |
| *Sebastes ruberrimus* | Yelloweye rockfish | Puget Sound - Georgia Basin DPS (ESA), Pacific Ocean inside waters population (SARA) | fish | T | 2011 | - | SC | 2011 | - |
| *Solidago houghtonii* | Houghton's goldenrod |  | plant | T | 1988 | 1997 | SC | 2006 | - |
| *Ursus maritimus* | Polar bear |  | mammal | T | 2008 | 2017 | SC | 2004 | - |

1. The species was grandfathered into the ESA.

2. Recovery of the species is considered not technically or biologically feasible at this time under SARA.

3. Humpback whales were recommended for downlisting to Special Concern by COSEWIC. This was gazette 30/06/2017; however, it is still relevant to assess the published Recovery Strategy.

4. Although likely extinct, this species is retained for inclusion in analysis.

5. Vancouver Island marmot is a foreign-listed species under the ESA.
