# Supplementary material for: Raising the bar: Recovery ambition for species at risk in Canada and the US
Source: PLoS One. 2019 Nov 19;14(11):e0224021. doi: 10.1371/journal.pone.0224021 (PMC6863564; doi:10.1371/journal.pone.0224021)
Supplement: S2 Table — Odds ratios and confidence intervals were obtained from Fisher’s Exact Tests. See Methods for further detail. Shading shows where the trend was opposite between the subset of paired data (n = 34) and the larger set of recovery documents (n = 208). (DOCX) [file pone.0224021.s002.docx]

Table S2. Comparisons of the quantitativeness and ambition of recovery goals by legislation, status, natural grouping, and habitat for species listed under both SARA and ESA (n=34). Odds ratios and confidence intervals were obtained from Fisher’s Exact Tests. See Methods for further detail. Shaded rows show where the trend was opposite between the subset of paired data (n=34) and the larger set of recovery documents (n=208). Odds Ratios in grey shading with italics have confidence intervals that overlap one (i.e., include the possibility of no difference in proportions).

| **Comparison** | | **Proportion of Goals (%)** | | **Odds Ratio (95% CI)** |
| --- | --- | --- | --- | --- |
| **Quantitativeness** |  | **Quant** | **Qual** |  |
| **of Ambition 4** | Overall | 28/37 (76) | 10/31 (32) | *6.3 (2.1-21.8)* |
| **Goals** | ESA | 18/23 (78) | 5/11 (45) | *4.1 (0.7-26.5)* |
| SARA | | 10/14 (71) | 5/20 (25) | 7.0 (1.3-46.7) |
|  | |  |  |  |
| **Legislation** | | **ESA** | **SARA** |  |
| Quantitative |  | 23/34 (68) | 14/34 (41) | *2.6 (1.0-9.1)* |
| Ambition 4 |  | 23/34 (68) | 15/34 (44) | *2.6 (0.9-8.0)* |
|  | |  |  |  |
| **Status** | | **Endangered** | **Threatened** |  |
| Quantitative | Overall | 26/47 (55) | 10/19 (53) | *1.1 (0.3-3.7)* |
| ESA | | 17/23 (74) | 6/11 (54) | *3.3 (0.6-5.7)* |
| SARA | | 9/25 (36) | 4/7 (57) | *0.4 (0.1-3.2)* |
|  | |  |  |  |
| Ambition 4 | Overall | 32/47 (68) | 6/19 (32) | *4.5 (1.3-17.5)* |
| ESA | | 18/22 (82) | 5/12 (42) | *5.9 (1.0-41.0)* |
| SARA | | 14/25 (56) | 1/7 (14) | *7.2 (0.7-374.9)* |
|  | |  |  |  |
| **Natural Grouping** | | **ESA** | **SARA** |  |
| Quantitative | Mammals | 8/10 (80) | 4/10 (40) | *5.4 (0.6-79.8)* |
| Birds | | 6/8 (75) | 5/8 (63) | *1.7 (0.1-29.0)* |
| Reptiles | | 1/2 (50) | 0/2 (0) | *Infinity* |
| Amphibians | | 0 | 0 | *NA* |
| Fish | | 1/3 (33) | 2/3 (66) | *0.3 (0-14.8)* |
| Invertebrates | | 1/3 (50) | 0/2 (0) | *Infinity* |
| Plants | | 8/10 (80) | 4/10 (40) | *3.6 (0.4-43.3)* |
| Ambition 4 | Mammals | 9/10 (90) | 3/10 (50) | *8 (0.6-470.1)* |
| Birds | | 6/8 (75) | 4/4 (50) | *2.3 (0.2-45.8)* |
| Reptiles | | 1/2 (50 | 0/2 (0) | *Infinity* |
| Amphibians | | 0 | 0 | *NA* |
| Fish | | 1/2 (50) | 2/3 (66) | *0.3 (0-14.8)* |
| Invertebrates | | 2/2 (100) | 0/2 (0) | *Infinity* |
| Plants | | 4/9 (44) | 4/9 (44) | *1 (0.1-9.2)* |
