# Supplementary figures and images for: Raising the bar: Recovery ambition for species at risk in Canada and the US
Source: PLoS One. 2019 Nov 19;14(11):e0224021. doi: 10.1371/journal.pone.0224021 (PMC6863564; doi:10.1371/journal.pone.0224021)

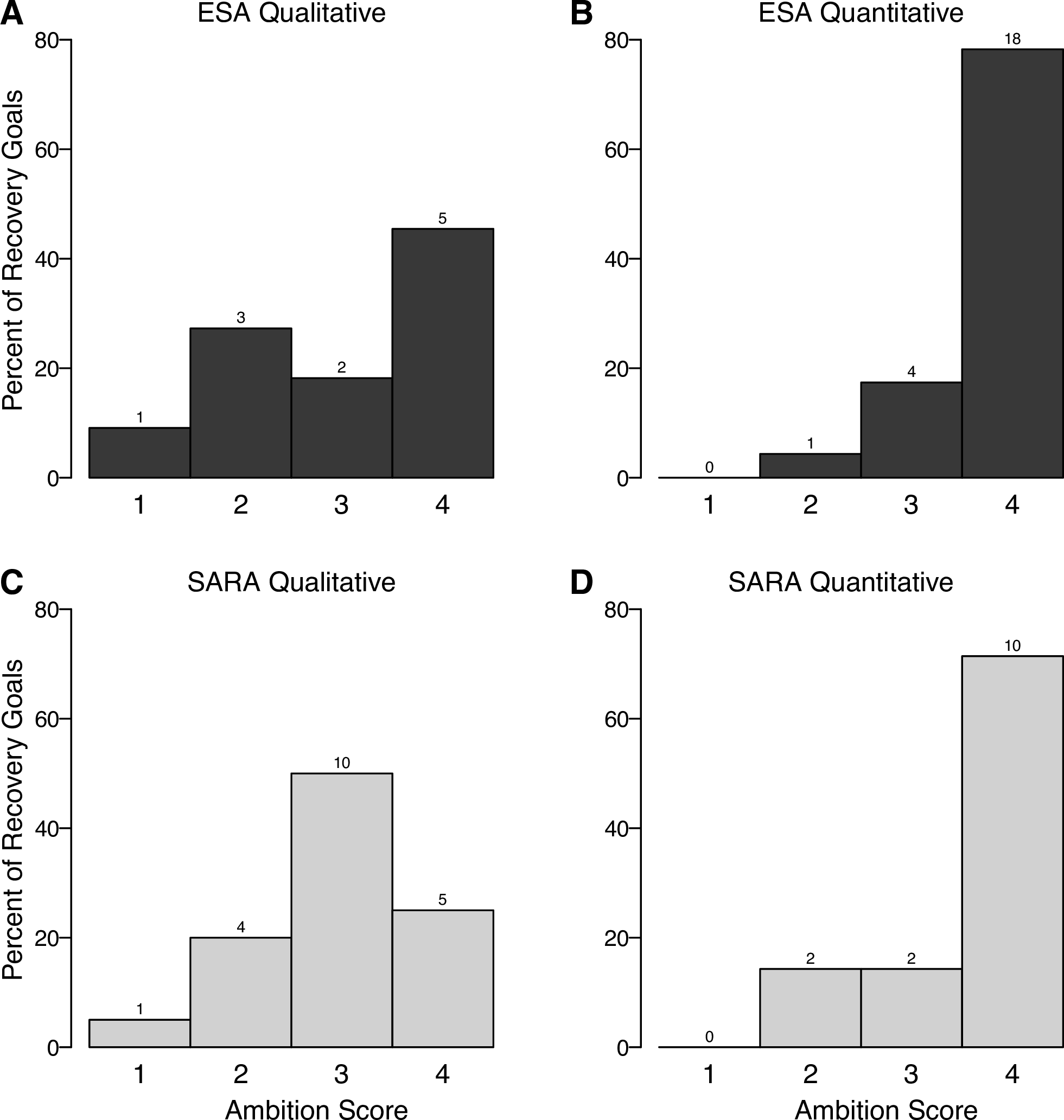

Supplement: S1 Fig — Bars show the percentage of recovery goals with ambition scores 1–4 for (A) qualitative goals under the ESA, (B) quantitative goals under the ESA, (C) qualitative goals under SARA, and (D) quantitative goals under SARA. Ambition of recovery goals within published recovery documents was scored on a scale from 1 to 5; however, no goals qualified for an ambition score of 5 (see Methods). The number of goals in each category is displayed above each bar. (TIF) [file pone.0224021.s003.tif]

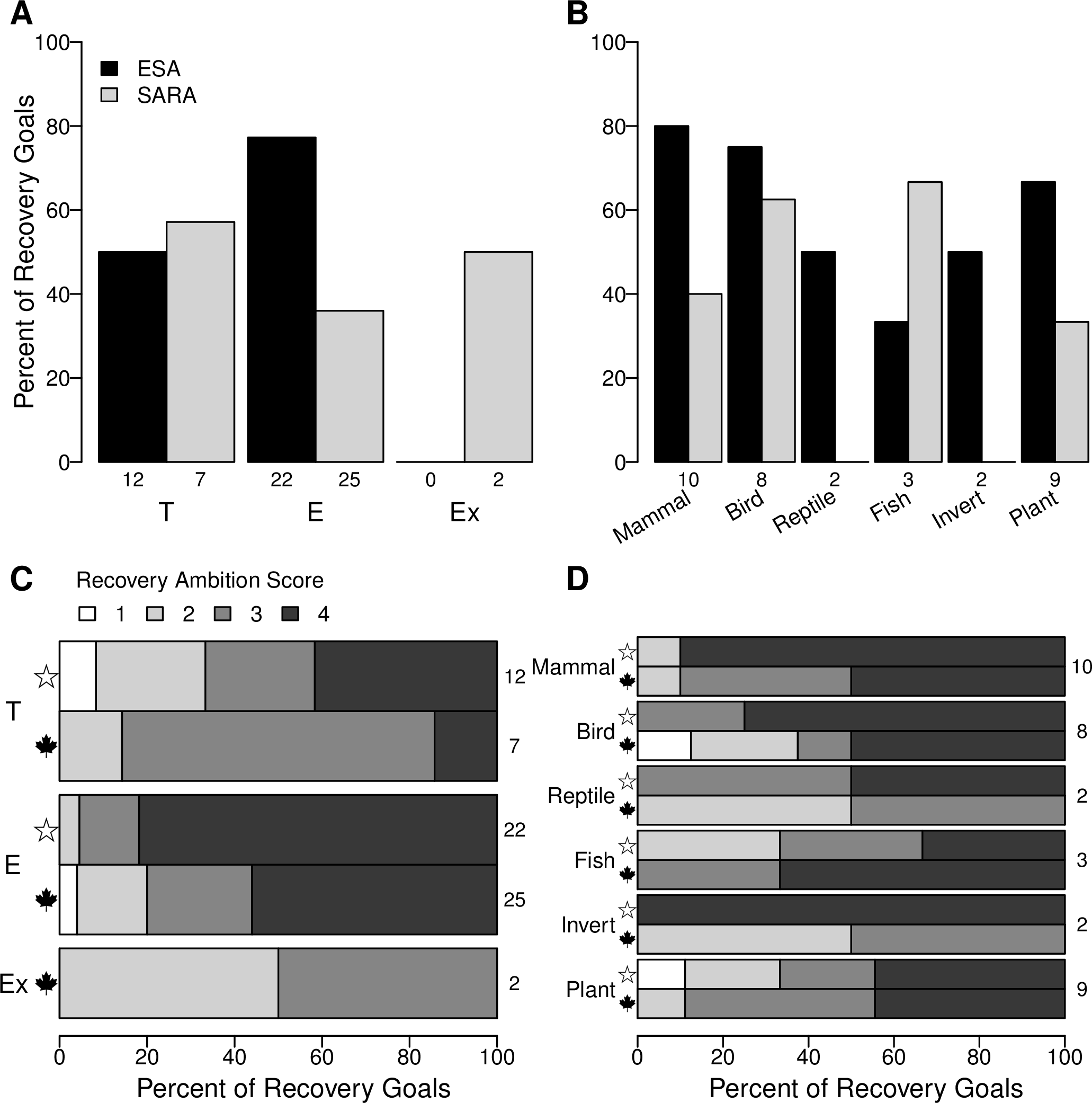

Supplement: S2 Fig — Percent of quantitative recovery goals (A) by species status (i.e., threatened [T], endangered [E], or extirpated [Ex]), and (B) by natural grouping. Ambition of recovery goals listed under the ESA (denoted by a star) and SARA (denoted by a maple leaf) (C) by status and (D) natural grouping. Ambition of recovery goals within published recovery documents was scored on a scale from 1 to 5; however, no goals received an ambition score of 5 (see Methods). Sample sizes are displayed to the right of bars. (TIF) [file pone.0224021.s004.tif]

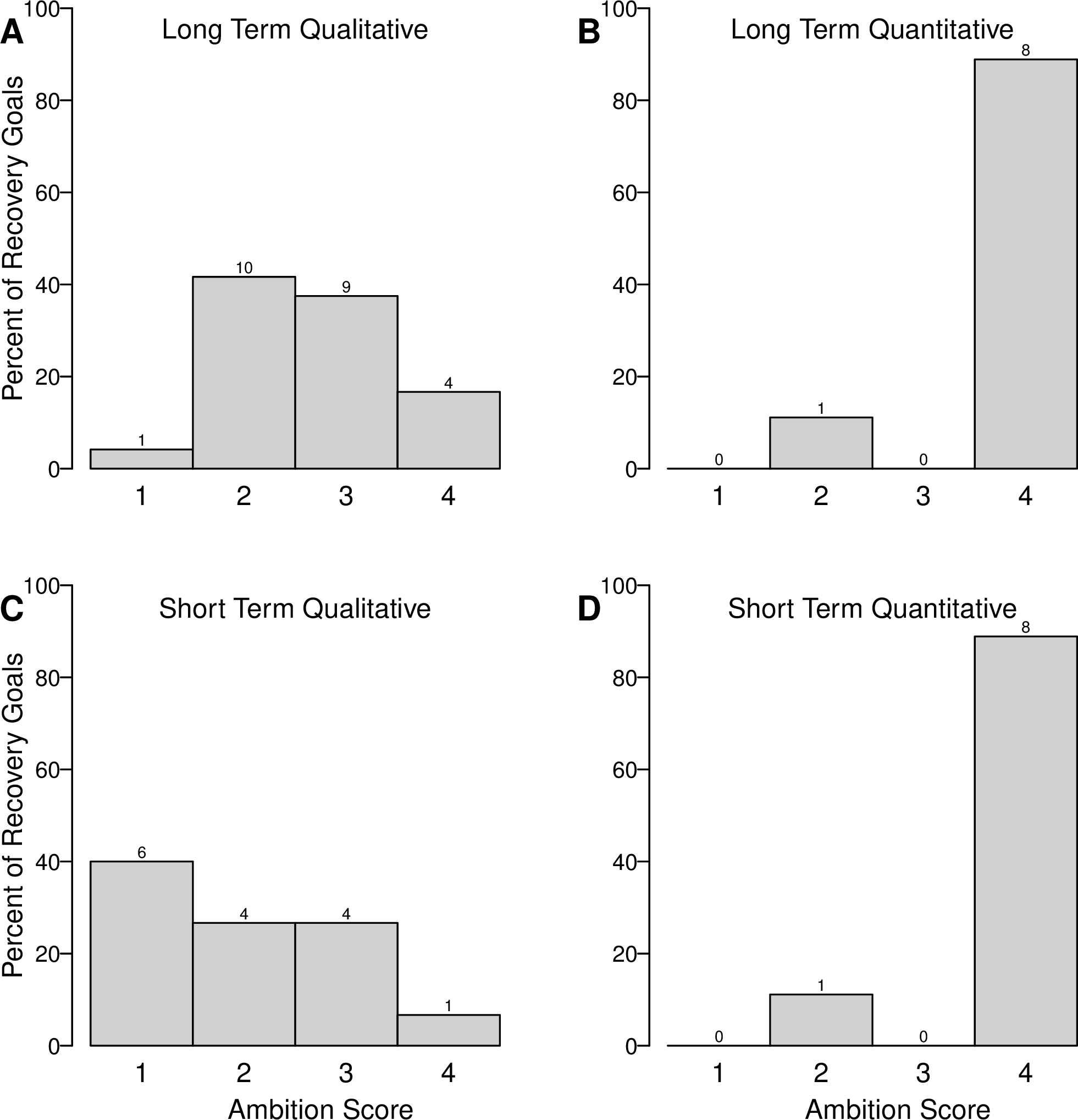

Supplement: S3 Fig — Bars show the percent of recovery goals with ambition scores 1–4 for (A) long-term qualitative goals, (B) long-term quantitative goals, (C) short term qualitative goals, and (D) short-term quantitative goals. Ambition of recovery goals within published recovery documents was scored on a scale from 1 to 5; however, no goals received an ambition score of 5 (see Methods). The number of goals in each category is displayed above each bar. Only species cross-listed under the United States’ Endangered Species Act are shown. Sample sizes are uneven because seven recovery plans contained only long-term goals and one strategy contained only short-term goals. (TIF) [file pone.0224021.s005.tif]
