# Supplementary material for: Raising the bar: Recovery ambition for species at risk in Canada and the US
Source: PLoS One. 2019 Nov 19;14(11):e0224021. doi: 10.1371/journal.pone.0224021 (PMC6863564; doi:10.1371/journal.pone.0224021)
Supplement: Attachment — Submitted filename: response letter outline-7.docx [file pone.0224021.s006.docx]

September 18, 2019

Re: Response Letter for PONE-D-18-01138

Dear Dr. Festa-Bianchet,

Please find attached our revised research article entitled ‘RAISING THE BAR: RECOVERY AMBITION FOR SPECIES AT RISK IN CANADA AND THE US’. Along with the revised manuscript, we have included our responses to the two reviewers. Please note that we are submitting our manuscript as a “new submission” as the deadline for re-submission has passed. Our submission was delayed due to the extra time required to collect new data as well as a variety of circumstances for co-authors (e.g., two co-authors on parental leave and all co-authors recently transitioned into new jobs or positions).

As recommended in the reviews, our revised manuscript includes additional data from species listed under endangered species legislation in Canada and the US, which has increased our sample size from 68 to 208. In addition, we changed the analyses to address Reviewer 2’s comments about co-linearity. Notably, our key results were strengthened by these new data and key findings remain similar to our previous version. We believe these major changes, in addition to other comments that have now been addressed, have greatly improved the manuscript. We are grateful to the Editorial team and to the reviewers for their thoughtful feedback and hope that the revised manuscript will be suitable for publication in PLOS ONE.

We look forward to hearing from you soon.

Sincerely,

Kylee Pawluk on behalf of Caroline Fox, Christina Service, Eva Stredulinsky, and Heather Bryan

**Reviewer Comments and Responses:**

Dear Dr. Pawluk,

Thank you for submitting your manuscript to PLOS ONE. After careful consideration, we feel that it has merit but does not fully meet PLOS ONE’s publication criteria as it currently stands. Therefore, we invite you to submit a revised version of the manuscript that addresses the points raised during the review process.

First, my apologies for how long this took - I had to contact 11 people to find 2 referees.

This is clearly an interesting manuscript, but I find that the reviewers raise several important and constructive criticisms, with which I agree.  Both reviewers question the rationale for only looking at species that are listed under both legislations.  That is because there is no evidence of pairwise comparisons in your paper (and Rev #3 correctly points out a possible non-independence of data point), and because this approach unnecessarily limits the sample size, as pointed out by both reviewers.  Rev#3 lists a series of statistical issues, which need to be addressed.  Inclusions of more species would clearly increase the power of these analyses, and I strongly encourage you to do so, partly because it is not obvious in this paper what is to be gained by only using cross-listed species.  Some careful matching by taxonomic group and possibly a couple of other criteria (harvested or not seems like an important one) would be better than limiting the analysis to cross-listed species.

**As recommended by both reviewers, we have scored additional recovery documents for species beyond our initial list of paired species. This has increased the sample size from 68 to 208. Additional species were randomly selected to ensure equal sample sizes by taxonomic group and legislation. However, since very few listed species are harvested, we did not include harvesting in our matching criteria.**

**We outline our criteria for including additional recovery documents on lines 202-208 of the manuscript.**

I am not sure about the point raised by Rev#3 about the differing mandate of specific agencies in the context of the current draft: presumably if a species is managed entirely by Parks Canada in Canada, it may be unlikely to make your list restricted to cross-listed species.  That may, however, become an important issue for a larger sample size: in Canada there is strong evidence that lead agency makes a difference, as bits of DFO remain somewhat hostile to SARA.

**This is an excellent point, and we certainly agree that the federal department or agency may influence recovery objective quality. While we remain open to this question, it was not part of the study’s objective. We would prefer to leave this component for a future study, perhaps one that focuses on only SARA-related challenges.**

Another important issue is that it is not clear in this paper, especially in the Introduction, what is the benefit of the comparative analysis for science or conservation practitioners: that needs to be better articulated.  Please also revise the last sentence of the Abstract to make it more explicit: what kind of guidance is provided by this study?

**We have revised the final sentences of the abstract with regard to specific guidance.**

“Combined, these findings provide guidance to strengthen recovery goals and improve subsequent conservation outcomes. In particular, species at risk planners should seek to attain higher recovery goal ambition, particularly for SARA-listed species, and include quantitative recovery goals wherever possible.” (line 37-40).

**We have also further elaborated on the value of a comparative (i.e., SARA and ESA) analysis for science and conservation practitioners.**

**“**By comparing across the two legislation types, our aim is to identify differences that could pinpoint areas for improvement as well as similarities that could identify weaknesses common to the development of quality recovery goals under both legislation types. Moreover, our analysis includes a subset of species that are cross-listed under both legislation types, the intent of which is to uncover cases that could benefit from increased cross-border sharing of information. Accordingly, study findings are intended to inform and provide guidance for the improvement of recovery goals in aid of species conservation.” (line 160-168).

Finally, one reviewer points out that referring to legal depositories is not quite up to standards for data availability, if you decide to resubmit a revised version please provide raw data files.

**Our data have now been uploaded to Dryad (**[**https://datadryad.org/review?doi=doi:10.5061/dryad.qk878ms**](https://datadryad.org/review?doi=doi:10.5061/dryad.qk878ms)**)**

We would appreciate receiving your revised manuscript by May 12 2018 11:59PM. When you are ready to submit your revision, log on to <https://pone.editorialmanager.com/> and select the 'Submissions Needing Revision' folder to locate your manuscript file.

**We apologize for the delayed re-submission; as noted above, the revision was prolonged due to collection of additional data and a variety of other circumstances, including two maternity leaves and changes in jobs/academic positions for all co-authors.**

If you would like to make changes to your financial disclosure, please include your updated statement in your cover letter.

**We have no changes to make at this time.**

To enhance the reproducibility of your results, we recommend that if applicable you deposit your laboratory protocols in [protocols.io](http://protocols.io/), where a protocol can be assigned its own identifier (DOI) such that it can be cited independently in the future. For instructions see: <http://journals.plos.org/plosone/s/submission-guidelines#loc-laboratory-protocols>

**We do not have a laboratory protocol for this work.**

Please include the following items when submitting your revised manuscript:

- A rebuttal letter that responds to each point raised by the academic editor and reviewer(s). This letter should be uploaded as separate file and labeled 'Response to Reviewers'.
- A marked-up copy of your manuscript that highlights changes made to the original version. This file should be uploaded as separate file and labeled 'Revised Manuscript with Track Changes'.
- An unmarked version of your revised paper without tracked changes. This file should be uploaded as separate file and labeled 'Manuscript'.

**We have included the required documents, including a response to reviewers below.**

We look forward to receiving your revised manuscript.

Kind regards,

Marco Festa-Bianchet
Academic Editor
PLOS ONE

Journal Requirements:

1.  When submitting your revision, we need you to address these additional requirements.
 
Please ensure that your manuscript meets PLOS ONE's style requirements, including those for file naming. The PLOS ONE style templates can be found at
<http://www.journals.plos.org/plosone/s/file?id=wjVg/PLOSOne_formatting_sample_main_body.pdf> and <http://www.journals.plos.org/plosone/s/file?id=ba62/PLOSOne_formatting_sample_title_authors_affiliations.pdf>

**We have consulted PLOS ONE’s style requirements to ensure the current version follows the template.**

2.  We note that you have indicated that data from this study are available upon request. PLOS only allows data to be available upon request if there are legal or ethical restrictions on sharing data publicly. For more information on unacceptable data access restrictions, please see <http://journals.plos.org/plosone/s/data-availability#loc-unacceptable-data-access-restrictions>.
 
In your revised cover letter, please address the following prompts:
 
a) If there are ethical or legal restrictions on sharing a de-identified data set, please explain them in detail (e.g., data contain potentially sensitive information, data are owned by a third-party organization, etc.) and who has imposed them (e.g., an ethics committee). Please also provide contact information for a data access committee, ethics committee, or other institutional body to which data requests may be sent.
 
b) If there are no restrictions, please upload the minimal anonymized data set necessary to replicate your study findings as either Supporting Information files or to a stable, public repository and provide us with the relevant URLs, DOIs, or accession numbers. For a list of acceptable repositories, please see <http://journals.plos.org/plosone/s/data-availability#loc-recommended-repositories>.
 
We will update your Data Availability statement on your behalf to reflect the information you provide.

**We have now uploaded our data to Dryad (https://datadryad.org/review?doi=doi:10.5061/dryad.qk878ms)**


[Note: HTML markup is below. Please do not edit.]

Reviewers' comments:

Reviewer's Responses to Questions

**Comments to the Author**

1. Is the manuscript technically sound, and do the data support the conclusions?

The manuscript must describe a technically sound piece of scientific research with data that supports the conclusions. Experiments must have been conducted rigorously, with appropriate controls, replication, and sample sizes. The conclusions must be drawn appropriately based on the data presented. 

Reviewer #1: Partly

Reviewer #3: No

2. Has the statistical analysis been performed appropriately and rigorously? 

Reviewer #1: Yes

Reviewer #3: No

3. Have the authors made all data underlying the findings in their manuscript fully available?

The [PLOS Data policy](http://www.plosone.org/static/policies.action#sharing) requires authors to make all data underlying the findings described in their manuscript fully available without restriction, with rare exception (please refer to the Data Availability Statement in the manuscript PDF file). The data should be provided as part of the manuscript or its supporting information, or deposited to a public repository. For example, in addition to summary statistics, the data points behind means, medians and variance measures should be available. If there are restrictions on publicly sharing data—e.g. participant privacy or use of data from a third party—those must be specified.

Reviewer #1: No

Reviewer #3: Yes

4. Is the manuscript presented in an intelligible fashion and written in standard English?

PLOS ONE does not copyedit accepted manuscripts, so the language in submitted articles must be clear, correct, and unambiguous. Any typographical or grammatical errors should be corrected at revision, so please note any specific errors here.

Reviewer #1: Yes

Reviewer #3: Yes

5. Review Comments to the Author

Please use the space provided to explain your answers to the questions above. You may also include additional comments for the author, including concerns about dual publication, research ethics, or publication ethics. (Please upload your review as an attachment if it exceeds 20,000 characters)

Reviewer #1: I have made many comments on the manuscript - minor in blue, major in awful pink. I enjoyed reading the manuscript, and found a lot of nuggets to contemplate, but could not find a good reason for this cross-listed species comparison. I believe all the major results and conclusions would flow from models of ambition in RS done separately in the two countries, which would increase the sample size considerably [note to the editor - this paper is based on only 34 paired documents]. This is not to say that a cross-listing comparison is not interesting, of course, but something does not line up. Please see my pink comments for a bit of elaboration.

I have recommended "minor revision" but this might end up being "major revision."

**We are grateful to reviewer 1 for these helpful comments. Most notably, we have now scored an additional 144 recovery documents, which has nearly tripled our previous sample size.**

Note also that the underlying data (the actual scores for the species from the documents) have NOT been made public - the authors blithely say that all the data are in the published RS presented by various governing bodies. Given that the scoring is subjective and the documents are long, this is bad form. The data must be included in the SM before this is published, anywhere.

**This is a good point. We have now uploaded the data to Dryad (https://datadryad.org/review?doi=doi:10.5061/dryad.qk878ms).**

Additional Comments from Reviewer 1 inserted into PDF file

Line 98: not clear why chapter and verse is cited for ESA, but only the Act for SARA when specific provisions are discussed.

**We have now modified these citations; both ESA and SARA citations are specific to the two Acts.**

Line 100: This is not actually true, is it? RS under SARA are supposed to delineate recovery objectives to “assist recovery and survival” (S41(1)d) - not quite the same as a “recovery goal.” This has been a bone of contention….

**We have modified this sentence to more broadly encompass the varying recovery objectives, whether to delist, assist recovery, or to recover species at risk.**

“Importantly, both acts require that Recovery Plans (ESA) and Recovery Strategies (SARA) be produced for species at risk [7,8]); these Recovery Plans and Strategies (hereafter “recovery documents”) outline what is required to assist recovery, recover and/or delist species, including the delineation of objectives for recovering species at risk.” (line 105-109).

Line 134: what does mismatched mean here? Between need and goal, between Canada and the US?

**We have modified this sentence for greater clarity.**

“Regardless of the drivers (political concerns, socioeconomic considerations, lack of information, and/or an absence of integrated conservation planning; e.g., [12]), varying quantitativeness and/or ambition of recovery goals in Canada and the US raises concerns over conservation effectiveness, efficiency, and ultimately, the persistence of at-risk species.” (line 142-146).

Line 143: Interesting at first glance, but we are not given any motivation as to WHY we should compare RS goals across the two countries.

**We have elaborated on why this comparison is being undertaken in the introduction (line 153-168). Short answer: (1) highly relevant for cross-listed species (i.e. species recoveries are co-dependent on the effectiveness of both countries legislation) and (2) via a contrast/comparison, there are opportunities for recovery goal quality improvement, perhaps particularly so for SARA, which is newer. Also, and perhaps most importantly, most SARA and ESA species are not recovering; improving conservation outcomes for listed species is a key priority, and a component of that improvement involves recovery goal quality.**

Line 149: thank you for being honest here.

Line 167: This is tricky - please clarify what is covered in (2) and (3) that is not covered in (1)? Ie, if the whole species or subspecies is listed in one jurisdiction, but only a DPS or DU is listed in the other, which criterion applies…(I wager this DPS DU thing drove you crazy when you were putting the data together. I would have thought that perhaps any WS with the same Latin binomial would be considered shared, independent of whether it was the whole jurisdictional range or just a subset, and then you could use taxonomic rank (DU/DPS, SSP, SP) as a predictor of ambition…

**We have now clarified that case 1) referred to species or subspecies with single recovery documents in each country whereas cases 2) and 3) referred to species where there were there were multiple recovery documents in both countries for different populations of the same species.**

“ESA and SARA cross-listed species were included in our analysis if: (1) they were identified as being the same species or subspecies with a single recovery document under each legislation or; (2) they represented the same population (e.g., white sturgeon, *Acipenser transmontanus*, Kootenay River population) with population-specific recovery documents under both legislation types or; (3) they were equivalent but spatially discrete populations (e.g., beluga whale, *Delphinapterus leucas*), or Designatable Units (DU, Canada; e.g., Sockeye salmon, *Oncorhynchus nerka*) or Distinct Population Segments (DPS, US; e.g., Atlantic salmon, *Salmo salar*) with population-specific recovery documents under both legislation types.” (line 180-189).

**As for including taxonomic rank as a predictor of ambition, the sample size in each category unfortunately would not allow such an analysis.**

Line 259: So, this paper is based on 34 * 2 documents. This must be considered borderline for quantitative analyses. We will need to see a lot of this visually to make sense of “effect sizes”

**We have now increased the sample size and have also retained the figures so patterns can be clearly visualized.**

Line 272: Given the small sample sizes here, I think raw numbers should be presented throughout, not %

**In addition to increasing the sample size, we have reported raw numbers and percentages in Tables 2 and S2.**

Line 279: Excellent - what I was looking for (referring to Figure 1—Ambition of quantitative and qualitative recovery goals)

**We have retained this figure, which now includes new data.**

Line 291: again, we need the raw numbers here

**Raw numbers are now included in addition to percentages in Tables 2 and S2.**

Line 366: “While….” :obscure sentence - not sure how RS and conservation gains are related…

**We have revised this sentence.**

“Endangered species legislation is essential to protect and recover at-risk species, but its successful implementation is inherently difficult, requiring considerable time, cost, and species-specific information. Previous assessments of national endangered species legislation and associated processes have identified multiple areas for improvement (e.g., listing process bias, critical habitat designation, and delays; [21,30]); recovery documents also offer a potentially rapid, strategic avenue for improving species’ conservation status [e.g., [29].” (line 392-398).

**Further, later in the discussion, evidence for a direct link between quantitative recovery goals (under ESA) and conservation outcomes is made.**

“One of our main findings was that only 38% of recovery documents assessed had recovery goals that were quantitative. This finding is highly relevant, as recovery goal quality may affect conservation outcomes. As a key example, Gerber and Hatch [29] found that quantitative recovery goals were associated with improved status of ESA-listed species. Further, quantitative recovery goals have been praised for providing consistency, efficiency, transparency, and legitimacy; consequently, recent reviews have called for their inclusion in recovery documents whenever possible [43]”. (line 414-421).

Line 374: Of course, one could look to see if Quantitative and Threat status predicted Ambition within SARA and ESA separately, increasing sample size. Again, the focus on cross-listed species is not well-motivated.

**We have substantially increased the sample size and type of analysis, including moving beyond cross-listed species.**

Line 387: This is an interesting result.

**Agreed, we think this is an interesting result as well.**

Line 390: indeed. Is there a way around this?

**We have modified the analysis, so that recovery goal quantitativeness is no longer a predictor of ambition and our original comment has been removed as it is no longer relevant. Our new approach involves an odds ratios analysis and we have modified this paragraph accordingly.**

“One of our main findings was that only 38% of recovery documents assessed had recovery goals that were quantitative. This finding is highly relevant, as recovery goal quality may affect conservation outcomes. As a key example, Gerber and Hatch [29] found that quantitative recovery goals were associated with improved status of ESA-listed species. Further, quantitative recovery goals have been praised for providing consistency, efficiency, transparency, and legitimacy; consequently, recent reviews have called for their inclusion in recovery documents whenever possible [43]. Despite these strengths, quantitative conservation targets have been criticized for their rigidity that may not be adaptive to complex ecological variation (e.g., climate change response) or socioeconomic criteria [44], for their application in situations when they are not biologically founded [45,46], and in cases where inherent uncertainty around targets is not reported [47]. When these concerns can be addressed, our results indicate that recovery goal quantitativeness should be improved where possible.” (line 414-427).

Line 399: This should be fleshed out. Also, “generally”

**We have modified this section to reflect our new analysis in response to reviewers’ comments. This sentence is no longer included in the manuscript.**

Line 400: possible explanation

**We have modified this section to reflect our new analysis in response to reviewers’ comments. This sentence is no longer included in the manuscript.**

Line 403: Why? Clarify please. The writing is getting a bit lazy here.

**This paragraph has been revised for clarity and to reflect or new analysis approach. See comments associated with line 390.**

Line 412: lazy wrting here

**This section has been revised for clarity and to reflect our modified analysis. See full revised paragraph under the comment associated with line 390.**

Line 419: This IS interesting, but this laundry list of findings and implications does not seem to be structured in any particular way.

**We have revised this paragraph for clarity and content.**

“Contributing new knowledge regarding the differences and similarities between ESA and SARA, this study also finds that SARA recovery goals are generally less quantitative and of lower ambition than those put forward under ESA. While recovery goal quality could be improved under both legislative acts, this appears to be particularly the case for SARA-listed species in Canada. Further, for the subset of species cross-listed under both ESA and SARA, there is evidence of varying goal quality between the two legislations, with SARA-listed species having lower recovery goal quality in general, in terms of both ambition and quantitativeness. Recovery goals with underwhelming ambition (i.e, ambition score 1 or 2) have been previously identified for SARA-listed species [17], but direct comparison with ESA species and the subset of cross-listed species has not been previously undertaken. Whereas McCune et al. [17] speculated that a species’ economic importance may influence recovery goal ambition, other factors may also contribute to reduced recovery goal quality under SARA.” (line 439-452).

Line 421: This, plus the work by Gerber and Hatch, points out that the present study has not found its place in the literature yet : what does it add to these studies and to the discussion on ES management generally?

**We have better emphasized the novelty of the present study in the concluding paragraph of the discussion.**

“In the midst of a global extinction crisis, effective conservation actions for recovering species at risk are crucial. When setting targets for at-risk species recovery, low quality recovery goals may undermine or stymie conservation efforts and, thus, are counterproductive. Given the potentially negative conservation outcomes associated with low quality goals, [e.g., [19], our results provide clear guidance for developing higher quality goals for recovery planning. Specifically, our findings highlight that recovery goals could be improved by focusing on the development of unambiguous, high-ambition (i.e., increasing population), and quantitative recovery goals wherever possible. Further, differences in recovery goal quality between ESA and SARA indicate that greater international information sharing [11] could provide a synergistic benefit in achieving conservation outcomes (e.g., via data and expertise sharing, joint recovery planning, and cross-border species management) for species cross-listed under both legislative acts. Ultimately, increasing recovery goal ambition for species with lower threat status could result in stronger conservation outcomes, although this topic warrants additional exploration. As a final point, attention to recovery goal quality is important because recovery documents are accessed by diverse users, including those without a scientific background and/or species-specific knowledge; by extension, recovery goals should be straightforward, explicit, and standalone. Although many other aspects of endangered species legislation require improvement, increasing recovery goal quality represents a strategic opportunity to effect meaningful and urgently needed conservation outcomes.” (line 492-514).

Line 433: These references suggest that this is a new comparison. Is it? If so, why 2007? These seem important numbers, and potential predictors of all sorts of things…

**Data from 2007 were the most recent financial data we were able to access for both countries. SARA is particularly challenging to get data on as three federal agencies are involved. 2007 was the last year that financial data were compiled from all agencies in Canada and accessible. Several emails were sent to the three Canadian Agencies involved in SARA work but we were not provided the information requested.**

Line 439: Several ideas in the same paragraph here…

**We have revised the entire discussion to reflect our modified analysis and to provide more clarity.**

Line 444: THIS seems imoprtant to explore - does the delisting requirement in the ESA actually drive more ambitious, more quantitiative recovery goals? That would be a proper prediction.

**Agreed that this is an interesting point to explore in a future manuscript.**

Line 471: And this ambition-quantitative connection is new to this study? Great if so, but this has little or nothing to do with cross-listed species.

**Manuscript has been substantially modified, with expansion to non-cross listed species. With a modified analysis, the ambition-quantitative connection is now less emphasized.**

Line 473: Really? I do not see how this flows from the rest of the paper…

**Content regarding international information sharing and communication has now been added to the introduction and better flows throughout the paper.**

“Moreover, our analysis includes a subset of species that are cross-listed under both legislation types, the intent of which is to uncover cases that could benefit from increased cross-border sharing of information.” (line 163-166)

“Relevant to potential information-sharing between countries, 18 of 34 species (53%) listed under both legislation types had goals that were quantitative under one legislation but not the other, including 13 cases where ESA goals were quantitative but SARA goals were not and 5 cases where SARA goals were quantitative and ESA goals were not.” (line 380-385).

“Further, differences in recovery goal quality between ESA and SARA indicate that greater international information sharing [11] could provide a synergistic benefit in achieving conservation outcomes (e.g., via data and expertise sharing, joint recovery planning, and cross-border species management) for species cross-listed under both legislative acts.” (line 500-505).

Reviewer #3: This is a very interesting study, which I found to be generally quite well written. It was very easy to follow in most places. The general topic of ambition in species recovery programs is also important.

**We appreciate this positive feedback.**

My major concern about the study surrounds the statistics. From some of the initial results, it appears that there is collinearity among the predictor variables. This makes interpreting the effects of these variables tricky, especially given the chosen information theoretic framework. There have been good critiques written about just this problem. As an example, in Table 2 the model with “legislation” is included in the interpreted models based in delta AIC. That’s OK in some circumstances (especially using the model to predict), but one way of interpreting this model is that it performs worse (in terms of AICc) than the model without this variable. This suggests that there is likely collinearity between legislation and threat status or quantitative goals, which we already know from previous results. Unfortunately, given the sample size, there is likely to be very little independent variation that can be attributed only to this (and perhaps other) variables. Given that the study is all about interpreting predictor variables, there are better techniques (variance partitioning) that can be used.

In addition, I was wondering how the species with the same identities were treated in the models. It appears that they may have been treated as independent data points, which is not the case. I also was wondering about the inherent relatedness in ambition and quantitativeness, which is acknowledged by the authors in the discussion. If these variables are inherently dependent, it’s really hard to test and interpret their statistical relationship. Finally, I think there may be an issue with overfit. Although there are six predictor variables, several are categorical (including one with six categories). Given the potential issue above with respect to species identity being non-independent, I can’t see how there isn’t an issue with overfit.

**We have considered the reviewer’s points about co-linearity and overfitting carefully. Co-linearity is an inherent problem with this type of analysis that will likely remain an issue even with our increased sample size. Moreover, the larger sample size may not entirely prevent overfitting. Finally, we agree that the modeling framework, particularly the use of quantitativeness as a predictor of ambition, adds more confusion than clarity to our analysis and interpretation. To address these issues, we have focused on univariate comparisons using Fisher’s Exact tests. This new analytical framework allows us to examine key variables of interest independently. It has also enabled us to shift the focus from quantitativeness as a predictor of ambition to quantitativeness as an additional and interesting measure of goal quality to ambition. These changes are reflected throughout the manuscript. Notably, our major findings and conclusions remain similar despite the new analyses.**

Given all the issues above, I was not 100% clear why only species represented in both countries were chosen. I suppose they can make for comparisons of how the same sort of problem is address in the US versus Canada, but to me this choice is not fully justified. If there are any major differences that are generalizable, would they not come out in comparisons among all lists?

**As described above, we have now examined additional species under both legislations. We have also further expanded on the rationale for comparing/contrasting recovery goals for species listed under SARA and ESA in the introduction and discussion.**

**Introduction:**

“Moreover, our analysis includes a subset of species that are cross-listed under both legislation types, the intent of which is to uncover cases that could benefit from increased cross-border sharing of information.” (line 163-168).

**Discussion:**

**“**Further, for the subset of species cross-listed under both ESA and SARA, there is evidence of varying goal quality between the two legislations, with SARA-listed species having lower recovery goal quality in general, in terms of both ambition and quantitativeness. Recovery goals with underwhelming ambition (i.e., ambition score 1 or 2) have been previously identified for SARA-listed species [17], but direct comparison with ESA species and the subset of cross-listed species has not been previously undertaken.” (line 466-473).

”Further, differences in recovery goal quality between ESA and SARA indicate that greater international information sharing [11] could provide a synergistic benefit in achieving conservation outcomes (e.g., via data and expertise sharing, joint recovery planning, and cross-border species management) for species cross-listed under both legislative acts.” (line 500-505).

Minor comments, with line #s where relevant:

Could it be that Action Plans (not tested here) have more quantitative goals in some circumstances?

**Good point — we’ve talked about this topic for a potential follow-up paper, but also note that a study of Action Plans would involve a substantially smaller sample size for SARA-listed species.**

I was also wondering about agency (especially in Canada). Some agencies have different cultures of producing documents, and Parks Canada, because of the limited geographic areas of its mandate, may have the luxury of better data and more quantitative goals.

**We certainly agree that agency, and the identification of potential agency-driven differences is a good question. We’d prefer to leave this topic for a subsequent study, but are open to suggestions.**

Lines 110-113 – This is a great example. Though it suggests to me that quantitative does not equate with achievement probability in any given plan (2.3% is very precise, but this population is surely not achieving this goal).

**This comment and associated 2.3% recovery goal refers to the southern resident killer whale example. With SRKW far from achieving 2.3% annual population growth, we would agree that a quantitative recovery goal does not necessarily equate with achievement probability in this particular case. However, this example raises the very difficult question of how/if recovery goals equate with actual recovery outcomes. We have tackled this still presently unanswerable, but excellent question in the discussion.**

“Ultimately, increasing recovery goal ambition for species with lower threat status could result in stronger conservation outcomes, although this topic warrants additional exploration.” (line 505-507).


Line 430 – the ESA numbers are highly skewed by a few species. I wonder if medians would be closer.

**Unfortunately, it is not possible with the financial data we were able to collect to calculate the median for SARA species. Therefore, it would not make sense to present the median for one legislation but not the other.**

6. If you would like your identity to be revealed to the authors, please include your name here (optional).

Your name and review will not be published with the manuscript. 

Reviewer #1: Arne Mooers

Reviewer #3: (No Response)
